# Putative Mitochondrial Sex Determination in the Bivalvia: Insights From a Hybrid Transcriptome Assembly in Freshwater Mussels

**DOI:** 10.3389/fgene.2019.00840

**Published:** 2019-09-13

**Authors:** Charlotte Capt, Sébastien Renaut, Donald T. Stewart, Nathan A. Johnson, Sophie Breton

**Affiliations:** ^1^Department of Biological Sciences, Université de Montréal, Montréal, QC, Canada; ^2^Centre de la Science de la Biodiversité du Québec, Université de Montréal, Montréal, QC, Canada; ^3^Department of Biology, Acadia University, Wolfville, NS, Canada; ^4^Wetland and Aquatic Research Center, U.S. Geological Survey, Gainesville, FL, United States

**Keywords:** comparative transcriptomics, mitochondrial DNA, hermaphroditism, ubiquitination, mitophagy, nucleases, methylation, doubly uniparental inheritance

## Abstract

Bivalves exhibit an astonishing diversity of sexual systems, with genetic and environmental determinants of sex, and possibly the only example of mitochondrial genes influencing sex determination pathways in animals. In contrast to all other animal species in which strict maternal inheritance (SMI) of mitochondria is the rule, bivalves possess a system known as doubly uniparental inheritance (DUI) of mitochondria in which maternal and paternal mitochondria (and their corresponding female-transmitted or F mtDNA and male-transmitted or M mtDNA genomes) are transmitted within a species. Species with DUI also possess sex-associated mtDNA-encoded proteins (in addition to the typical set of 13), which have been hypothesized to play a role in sex determination. In this study, we analyzed the sex-biased transcriptome in gonads of two closely-related freshwater mussel species with different reproductive and mitochondrial transmission modes: the gonochoric, DUI species, *Utterbackia peninsularis*, and the hermaphroditic, SMI species, *Utterbackia imbecillis*. Through comparative analysis with other DUI and non-DUI bivalve transcriptomes already available, we identify common male and female-specific genes, as well as SMI and DUI-related genes, that are probably involved in sex determination and mitochondrial inheritance in this animal group. Our results contribute to the understanding of what could be the first animal sex determination system involving the mitochondrial genome.

## Introduction

Only one putative sex determination system involving mitochondria and their genome (mtDNA) has been reported in animals (Breton et al., 2011; Breton et al., 2018). It implicates the unorthodox system of doubly uniparental inheritance (DUI) of mitochondria in bivalve molluscs, which is also the only known exception to strict maternal inheritance (SMI) of mitochondria in animals (Birky, 2001; Breton and Stewart, 2015). Over 100 DUI species have been reported to date (Gusman et al., 2016). In these taxa, both maternal and paternal mitochondria (and their corresponding female-transmitted or F mtDNA and male-transmitted or M mtDNA) are transmitted uniparentally through eggs and sperm, respectively (see Breton et al., 2007; Passamonti and Ghiselli, 2009; Zouros, 2013 for reviews). In all DUI species, the F mtDNA is found in female gonads and in the somatic tissues of both sexes, whereas the M mtDNA is found in sperm and is sometimes detected in small quantities in male soma (see Breton et al., 2017b for a review). The M and F mtDNAs can be extremely divergent, with up to 40% intraspecific DNA divergence (Doucet-Beaupré et al., 2010; Guerra et al., 2017). The mechanism(s) by which such molecular divergence of mtDNAs is tolerated within a species are of considerable interest considering that heteroplasmy, i.e. the presence of two or more different mtDNAs within an organism, can cause severe disease or death in humans (Picard et al., 2016; Wallace, 2016).

Species with DUI also possess sex-associated mtDNA-encoded proteins (in addition to the typical set of 13) that are not apparently homologous to any other known genes (i.e. they are mitochondrial ORFans) (Breton et al., 2009; Breton et al., 2011; Milani et al., 2013a). Specifically, the F mtDNA contains the F*-orf* gene and the M mtDNA contains the M*-orf* gene, both of which are relatively conserved across species within a family (Breton et al., 2009; Breton et al., 2011; Milani et al., 2013a; Mitchell et al., 2016). In freshwater mussels (Unionida), gonochorism is perfectly correlated with the presence of DUI and these novel sex-specific proteins, whereas hermaphroditic species lack the M mtDNA (i.e. they possess SMI of mtDNA) and have macromutations in the region of their mtDNA genome that is homologous to the F*-orf* gene of the F mtDNA in their gonochoric relatives (Breton et al., 2011; Breton et al., 2014). In keeping with the transition from gonochorism to hermaphroditism, the mitochondrial genome present in these species is referred to as an H mtDNA and the gene of interest is known as an H-*orf* (Breton et al., 2011). These mtORFans have been hypothesized to be part of the sex determination system (Breton et al., 2011), and if true, this would make DUI the first animal sex determination system involving the mtDNA (heteromorphic sex chromosomes are absent in bivalves). However, we are still far from fully understanding the underlying link between DUI and sex determination.

A first transcriptomic study of mature male and female gonads of the DUI clam *Ruditapes philippinarum* proposed a relationship between sex, mitochondrial inheritance, and ubiquitination (Ghiselli et al., 2012). Ubiquitination is well known for being involved in the elimination of paternal mitochondria in mammals (Sutovsky et al., 1999). It is also known to play a role in sex determination in *Caenorhabditis elegans* and *Drosophila* (Ghiselli et al., 2012). Ghiselli et al. (2012) found a relationship between sex and differential expression of several ubiquitination genes in *R. philippinarum*, making them likely candidate genes for the regulation of sex‐specific aspects of the DUI system. Following this work, Milani et al. (2013a) focused on three of these genes that showed the most significant transcriptional bias and the highest levels of transcription (baculoviral IAP repeat‐containing 4 [*birc*]; proteasome subunit alpha 6 [*psa*]; AN1 zinc finger ubiquitin‐like domain [*anubl1*]). They hypothesized that these genes could play a role in sex determination and be responsible for the maintenance or degradation of sperm mitochondria during embryo development in DUI species. In addition, Capt et al. (2018) hypothesized that if a modification of the ubiquitination mechanism is responsible for the retention of the paternal mtDNA in male bivalves, then molecular signatures of this modification should be discernable in all DUI species. The same three differentially expressed candidate genes were indeed identified in their comparative analysis of male and female gonad transcriptomes for the two DUI freshwater mussel species *Venustaconcha ellipsiformis* and *Utterbackia peninsularis* (Capt et al., 2018). This study also revealed that DNA methylation could be involved in the regulation of DUI (Capt et al., 2018) and/or in sex determination and differentiation, as seen in other species (Liu et al., 2015; Capt et al., 2018).

Ubiquitination is not the only process involved in SMI of mitochondria in animals. The mechanisms underlying SMI are widely variable and suggest recurrent loss and restoration and/or several independent origins of this nearly ubiquitous phenomenon (Birky, 2001; Breton and Stewart, 2015). For example, paternal mtDNA can be eliminated by a nuclease-mediated mechanism either during spermatogenesis or after fertilization, or paternal mitochondria can be selectively degraded by autophagy after fertilization (Sato and Sato, 2017). To better understand the molecular basis of DUI, Punzi et al. (2018) selected classes of proteins known to be involved in the maintenance of SMI and compared their features in two closely-related gonochoric clam species differing in their mitochondrial inheritance mechanism, that is, the SMI species *Ruditapes decussatus* and the DUI species *R. philippinarum*. Their analysis of gonad transcriptomes focused on nucleases and polymerases, ubiquitination, and proteins involved in autophagy/mitophagy. Their results provided no evidence for a role of nucleases/polymerases or autophagic machinery in the enforcement of SMI in *R. decussatus*, but transcripts of ubiquitinating enzymes were retrieved, providing additional support to previous transcriptomic studies (Punzi et al., 2018).

In the present study, we take advantage of the existence of closely-related gonochoric–DUI and hermaphroditic–SMI freshwater mussel species to better understand the genetic mechanisms that govern DUI and sex determinations in bivalves. Transcriptomic analyses were performed on three types of gonads, which will also be referred to as “sexes”, i.e. hermaphroditic gonad samples of *Utterbackia imbecillis* and available male and female gonadal transcriptome data from the species *U. peninsularis* (Capt et al., 2018). We produced a hybrid assembly that included all libraries from both species, which was used as a reference to map the sequencing reads of each sex. Male–female (MF), hermaphrodite–female (HF) and hermaphrodite–male (HM) comparisons were performed to identify candidate genes involved in sex determination and/or mitochondrial inheritance. In light of information from previous studies (Ghiselli et al., 2012; Milani et al., 2013a; Capt et al., 2018; Punzi et al., 2018), particular attention was given to the following gene ontology (GO) categories to identify candidate genes putatively involved in sex determination or mitochondrial inheritance: reproduction, ubiquitination, nucleases, autophagy/mitophagy and methylation.

## Methods

### Sample Collection and RNA Extraction

Adult *U. imbecillis* mussels used for this study were collected in the Suwannee River (Florida, USA; lat 29.58684, long －82.94095) in July 2014. All mussels were kept alive during their shipment to the Université de Montréal (QC, Canada). Mussels were dissected and gonads inspected under the microscope. Only mature gonads were kept in the current study and frozen at －80°C until further analyses.

Total RNA extraction from frozen gonad tissues was performed as described in Capt et al. (2018) on eight *U. imbecillis* samples. Quality and quantity of extracted RNA was assessed *via* electrophoresis on a 1% agarose gel and using a BioDrop μLITE spectrophotometer; one sample with an absorbance reading A260/A280 below two was discarded (i.e. seven “high quality” RNA samples remained). Because *U. imbecillis* specimen can be confused visually with *U. peninsularis* (both species can be found in this sampling site), that these samples were indeed hermaphrodites was validated by verifying the presence of the mitochondrial hermaphrodite open reading frame (*h-orf*) specific to *U. imbecillis* (Breton et al., 2011) by PCR (primer sequences: UttimbHorf_F 5’-ATCTTTCTAATTAAGTGTATACG-3’ and UttimbHorf_R 5’-TTGGGCTAGGTTTGTTACGG-3’) and sequencing. Three PCR-confirmed *U. imbecillis* samples were sent to the Institute for Research in Immunology and Cancer Institute (IRIC, Université de Montréal, Montréal, Canada) for Illumina library preparation (mRNA-Seq) and paired-end (2 × 100 bp) sequencing (Illumina HiSeq2000; San Diego, CA). Paired-end sequencing was chosen for these three samples (no replicate) because it increases the quality of *de novo* assembly in non-model species and allows for more accurate isoform analyses (Conesa et al., 2016). The same procedure was used in Capt et al. (2018) for *U. peninsularis* (four female and four male gonad samples without replicates).

### Reads Quality Assessment, *De Novo* Assemblies and Completeness Appraisal

Paired-end reads were trimmed with Trimmomatic (v0.32; Bolger et al., 2014) with the options ILLUMINACLIP : TruSeq3-PE.fa:2:30:10 LEADING:3 TRAILING:3 SLIDINGWINDOW:6:10 MINLEN:36. Illumina paired-end adaptors were removed and trailing and leading sequence ends were trimmed if quality (Phred score) dropped below 30. If mean quality dropped below 10 in a six base wide sliding window, nucleotides were replaced by N, and finally, if a trimmed read dropped below a length of 36bp, both paired-end reads were removed. The quality of reads was controlled and visually inspected with FASTQC (v0.11.2; Andrews, 2010), as described in Capt et al. (2018).

Two distinct *de novo* assemblies were performed with the trimmed reads for each species, using Trinity (Haas et al., 2013). The analytical pipeline used to obtain the transcriptome of *U. imbecillis* and the main reference transcriptome, which is referred to as the “hybrid” transcriptome for the rest of the study, is the same as the one previously described for *U. peninsularis* (Capt et al., 2018), i.e. with a minimum length fixed at 300pb for each contig, and other parameters left as default. This was done to allow TransDecoder to recognize Open Reading Frames (ORFs) and reduce the rate of false positives (https://transdecoder.github.io/). The hybrid transcriptome was obtained with the reads of eleven individuals (i.e. our three *U. imbecillis* hermaphrodites and four male and four female *U. peninsularis*), and allowed for differential gene expression analysis among the three sexes to avoid bias that would show up from comparing two different transcriptomes. A similar approach was used to study head transcriptomes of two fruit fly species of the genus *Anastrepha* (Rezende et al., 2016). Most analyses were thus performed on the hybrid transcriptome whereas the gonochoric (*U. peninsularis*) and the hermaphrodite (*U. imbecillis*) transcriptomes were used to assess the validity of the hybrid transcriptome, or to check for the presence of species-specific genes. The hybrid transcriptome underwent a clustering of contigs that shared >90% similarity, using CD-HIT-EST (v4.6; Li and Godzik, 2006). This step was added to avoid having multiple orthologous copies of genes from *U. imbecillis* and *U. peninsularis* in the hybrid assembly, and consequently false-positives in differential gene expression analyses. The cutoff choice was based on the divergence between mitochondrial female and hermaphrodite nucleotide sequences.

The degree of completeness of the transcriptomes was assessed with Benchmarking Universal Single-Copy Ortholog (BUSCO) software (busco_v3, mode -tran). Two core gene databases, metazoa (978 genes) and eukaryota (303 genes), were used and compared with the Trinity-based *de novo* assemblies. This comparison measures the presence of near-universal highly conserved single-copy orthologs, and provides information about the completeness of the transcriptomes based on what is expected, depending on the database (Simão et al., 2015).

### Reads Alignment and Differential Gene Expression

Raw reads from each individual were aligned to the reference hybrid transcriptome using Bowtie2 (v2.2.4; Langmead and Salzberg, 2012) with default parameters. Next, reads were sorted, indexed, and the corresponding numbers of mapped reads by contig were extracted with Samtools software (v1.1; Li et al., 2009), following the pipeline in Capt et al. (2018). Results were normalized based on Robinson and Oshlack (2010). Given that we previously observed that both DESeq2 (Love et al., 2014) and edgeR (Robinson and Oshlack, 2010) provide similar gene expression patterns (Capt et al., 2018), only edgeR (version edgeR_3.12.0) was used in the current study to simplify analyses and interpretation. EdgeR selects for a more restricted number of differentially expressed genes (DEGs) and increases identification of true positives (Soneson and Delorenzi, 2013; Zhang ZH et al., 2014). DESeq2 was used only to acquire fragments per kilobase of exon per million fragments sequenced (FPKM) values, using “robust = F” to sum the raw counts. DEGs were obtained with edgeR by selecting all contigs with a FPKM value greater than 1 and a fold-change ≥1 or ≤ －1 in one of the three sexes, for each paired-comparison. Gene expression comparisons were performed two per two between hermaphrodites and males (HM), hermaphrodites and females (HF), and males and females (MF). To do so, contrast was fixed at 1 for one sex for each comparison, －1 for the other, and 0 for the sex absent in the comparison, and no intercept was given for the design because there was no ‘control’ value in the current design.

A False Discovery Rate (FDR) adjustment (Benjamini and Hochberg, 1995) was performed to account for multiple comparisons; only values with a corrected p-value < 0.05 were kept as DEG. Unigenes that were expressed only in one sex, i.e. with a FPKM value >1 in one sex and a FPKM value of 0 for the two others, were considered as sex-specific. When genes were hermaphrodite-specific, the associated annotation was checked in the *U. imbecillis* transcriptome to confirm its validity and specific role in hermaphrodites. A Principal Component Analysis (PCA) was also performed on regularized log transform (rlog) values of the most expressed genes, to verify if individuals were clustering according to their sex.

### Annotations and Gene Ontology Enrichment

Assembled contigs from each transcriptome were searched against protein and nucleotide sequences databases. First, potential ORFs, and their linked protein-coding sequences, were predicted using TransDecoder (r20140704; Haas and Papanicolaou, 2017). If several ORFs were found in one contig, the longest was selected. This new TransDecoder list of ORFs allowed a BLASTp search (*e*-value <1e－5) against protein databases (Swiss-prot and TrEMBL [uniref90 cluster]) available in the Universal Protein Resource (UniProt, April 2018). Swiss-prot is a high quality, manually annotated and non-redundant protein sequence database that focuses mainly on model species to ensure quality annotation. Uniref90 provides computationally generated annotations unfound in Swiss-prot and allows a higher annotation success, but the database is not manually curated. To maximize the hybrid transcriptome annotation success, an additional BLAT search (BLAST-like alignment tool) was performed against NCBI non-redundant (NR) protein database. A BLASTx search (*e*-value <1e－5) on unigene sequences against Swiss-prot nucleotide database was also performed to complete the annotation [this additional step was also done for *U. peninsularis* (Capt et al., 2018).

Gene ontology enrichment analyses were performed by combining annotations and differential gene expression information using GOseq (v1.28.0; Young et al., 2010). After FDR correction on *p*-values, GO terms significantly over-represented were identified with a FDR corrected *p*-value <0.05.

## Results and Discussion

### Transcriptome Assemblies and Completeness

A total of 188,337,818 paired-end reads was produced for the three *U. imbecillis* samples and 348,652,616 for male and female *U. peninsularis* samples (Capt et al., 2018), giving a total of 536,990,434 paired-end reads for both species. The FASTQ raw sequences are accessible under accession numbers SRR8782945-7 (*U. imbecillis*) and SRR6279376-83 (*U. peninsularis*; Capt et al., 2018) in the National Centre for Biotechnology Information (NCBI) Sequence Read Archive (SRA) database.

After the trimming process, 439,968,848 high-quality raw reads (81.9%) were retained to build a hybrid assembly of 215,908 transcripts (after the 90% clustering with CD-HIT; before this step, the hybrid assembly provided 245,596 transcripts). The final hybrid transcriptome had an average contig length of 798 bp and a N50 of 1,034. The number of transcripts and median contig length were similar to the values obtained for *U. peninsularis* (Capt et al., 2018). Complete statistics for all transcriptomes are available in **Table 1**.

**Table 1:** *De novo* assembly quality and annotation statistics of *Utterbackia imbecillis, Utterbackia peninsularis* and the hybrid transcriptomes.

	Hybrid	*U. imbecillis*	*U. peninsularis* (Capt et al., 2018)
Assembly statistics			
Total raws	536,990,434	188,337,818	348,652,616
Average raws	44,749,203	47,084,454	43,581,577
Total trimmed raws	439,968,848	151,648,110	288,320,738
Average trimmed raws	36,664,071	37,912,028	36,040,092
Total trinity ‘genes’	197,379	111,958	165,788
Total trinity transcripts	215,908	130,160	200,961
Percent GC	34.71	35.23	33.36
Contig N10	4,222	5,326	5,581
Contig N20	2,883	3,724	3,895
Contig N30	2,123	2,804	2,916
Contig N40	1,551	2,144	2,224
Contig N50	1,128	1,623	1,655
Median contig length	497	557	558
Contig N40	834.10	1,015.56	1,750
Total assembled bases	180,089,785	132,185,742	206,277,984
Contig N10	4,124	4,765	4,882
Contig N20	2,794	3,278	3,298
Contig N30	2,014	2,430	2,409
Average contig length	1,444	1,808	1,026.46
Contig N50	1,034	1,333	1,251
Median contig length	482	516	510
Average contig	798.47	904.79	884
Total assembled bases	157,600,779	101,298,523	146,628,566
Annotations statistics			
TransDecoder	30,964	31,679	40,360
Swiss-prot–BLASTp	26,648/215,908 (12.3%)	20,683/130,160 (15.9%)	26,469/200,961 (13.1%)
Uniref90–BLASTp	32,430/215,908 (15.0%)	24,982/130,160 (19.2%)	32,515/200,961 (16.2%)
NCBI NR–BLATp	19,274/215,908 (8.9%)	–	–
Swiss-prot–BLASTx	21,338/215,908 (9.9%)	–	28,495/200,961 (14.2%)

**Supplementary Figure S1** shows the results of the completeness assessment performed using BUSCO. This analysis resulted in 97.8% completely recovered genes for *U. imbecillis* (20.6% duplicated, 1.6% fragmented, 0.6% missing), and 98.4% completely recovered genes for the hybrid transcriptome (32.2% duplicated, 0.6% fragmented, and 1% missing). Similar results were also obtained for *U. peninsularis* (Capt et al., 2018). These results validated the completeness of our assemblies and allowed further and reliable analyses of gene expression from the hybrid transcriptome.

### Annotation

Transdecoder recognized 30,964 ORFs from the hybrid transcriptome, which were used to perform BLASTp searches against Swiss-Prot and Uniref90, and BLAT searches against NCBI NR. A total success annotation of 12.3, 15, and 9% were respectively obtained with these three approaches (**Table 1**). BLASTx against Swiss-prot annotated 9% of the unigenes. Most hits came from mollusk species (data not shown), particularly from the oyster *Crassostrea gigas* for which whole-genome sequencing and annotation were recently completed (Zhang et al., 2012). When compared with the the recently reported genomes for the freshwater mussels *Venustaconcha ellipsiformis* (Bivalvia Unionida: Renaut et al., 2018) and *Limnoperna fortunei* (Bivalvia: Mytilidae; Uliano-Silva et al., 2018), both annotated and assessed for their completeness using the same approach that we did (i.e., respectively with Benchmarking Universal Single-Copy Ortholog [BUSCO] software and BLASTing against uniprot and NCBI NR), our results with BUSCO were comparable and even a bit better [81.9–85% of complete BUSCOs for Renaut et al. (2018) and Uliano-Silva et al. (2018) and 97.7% complete BUSCOs for the present study]. Similarly, Uliano-Silva et al. (2018) annotated 26,198 out of 60,717 predicted protein-coding genes (43%) and Renaut et al. (2018) annotated around 39% of predicted protein genes (>300bp) using BLAST against Uniprot and, for example, we annotated 24,982 of our *U. imbecillis* transcripts (out of 130,160 = 19.2%). Our percentage of annotation is rather low because we considered all our transcripts (as in other transcriptomic studies). However, if we consider only ORFs identified by Transdecoder (i.e., 31,679 ORFs >300bp from our transcriptome), our percentage of annotation would have been comparable to Uliano-Silva et al. (2018) and Renaut et al. (2018). Also, our results were similar to what was obtained for the gonadal transcriptome of *U. peninsularis* (Capt et al., 2018). Success annotation in *U. imbecillis* was also similar to what was obtained for *U. peninsularis* and the hybrid transcriptome (**Table 1**). Very few hits came from bacteria or trematodes species, indicating that our gonad samples were not contaminated. Overall, our results are similar to those reported in other transcriptomics studies of bivalve species (Shi et al., 2015; Patnaik et al., 2016; Yarra et al., 2016; Wang et al., 2017), and confirm the difficulty to annotate non-model species, given that few well-characterized model organisms are closely related to bivalves.

### Screening of Differentially Expressed and Sex-Specific Genes

**Figure 1:**
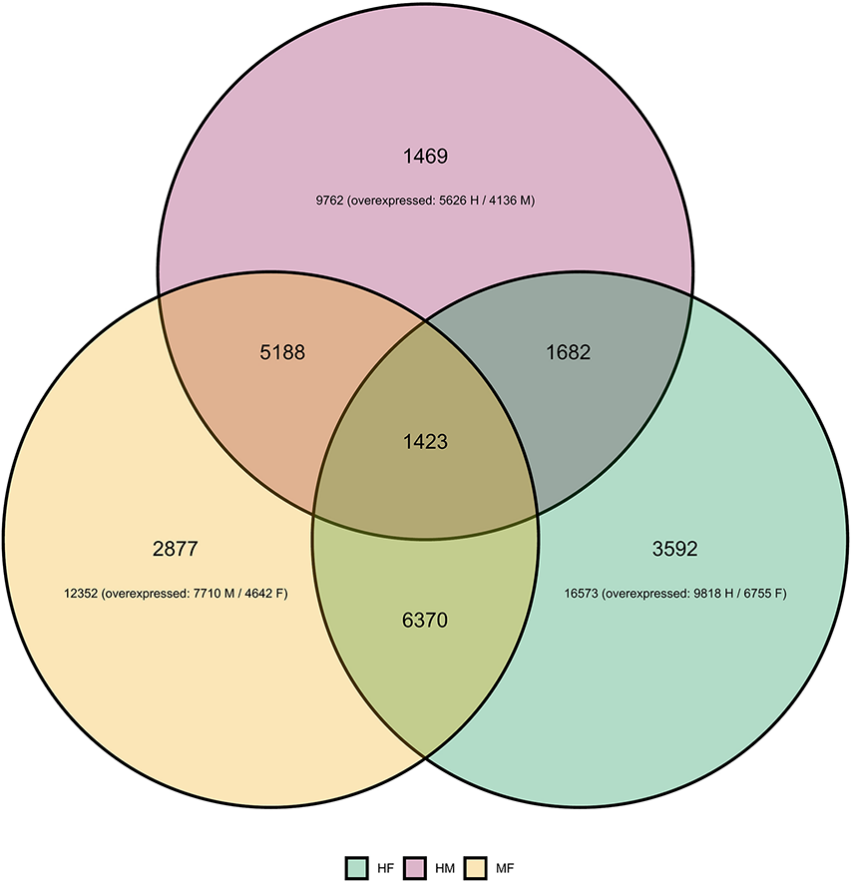
Differential expression analysis of *Utterbackia* spp unigenes. Venn diagram shows differentially expressed genes (DEGs) between sexes: hermaphrodite–female (HF in green), hermaphrodite–male (HM in pink) and male-female (MF in orange) comparisons. The figure shows the number of DEGs unique for one comparison (e.g. 2,877 for MF), the number of DEGs shared between two comparisons (e.g. 5,188 between MF and HM comparisons) and the number of DEGs shared by all comparisons (1,423).

**Figure 2:**
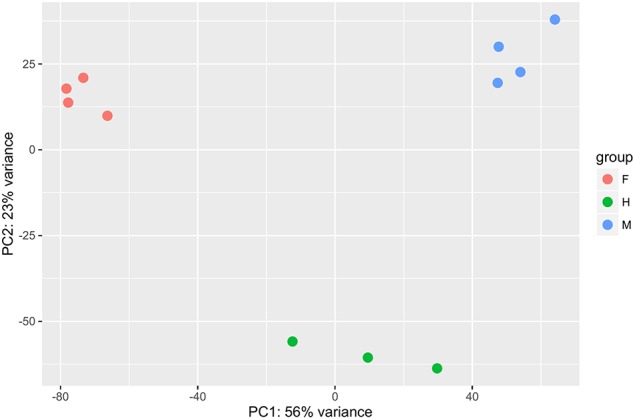
Principal Component Analysis (PCA) performed on regularized log transform (rlog) values of the first thousand most expressed genes per pairwise comparison between the three sexes (males, females and hermaphrodites). The four males are shown in blue, the four females in red and the three hermaphrodites in green.

Differential gene expression analyses showed that the highest number of differentially expressed genes (DEGs) was observed between hermaphrodites and females (16,573 for the HF comparison), with 9,818 genes up-regulated in hermaphrodites and 6,755 in females (**Figure 1**). We found 12,352 DEGs between males and females (MF comparison), with 7,710 genes up-regulated in males and 4,642 in females. Finally, 9,762 genes were differentially expressed between hermaphrodites and males (HM comparison), with 5,626 genes up-regulated in hermaphrodites and 4,136 in males. A higher number of DEGs in hermaphrodites is in agreement with previous studies (e.g. Shi et al., 2018), and can be explained by the production of both gametes in hermaphrodite gonads. **Figure 1** shows the overlap in DEGs for each comparison. For example, for the HF comparison 16,573 DEGs were found, 6,370 of which were also DEGs in the MF comparison, 1,682 were also DEGs in the HM comparison, and 1,423 were DEGs in all comparisons. The Principal Component Analysis (PCA) performed on regularized log transform (rlog) values of the most expressed genes confirmed that individuals clustered according to their sex (**Figure 2**), reinforcing the reliability of our results and suggesting low biological variability among samples of the same sex.

To unravel potential candidate genes involved in sex determination and/or DUI in *Utterbackia* spp., we first focused on sex-specific genes (i.e. those expressed only in one sex and not in the two others). **Table 2** shows sex-specific genes based on FPKM values. Our analyses revealed 567 genes specific to hermaphrodites, only eleven of which were annotated. DUI individuals showed fewer sex-specific genes with 59 for males (7 annotated) and 100 for females (10 annotated) (**Table 2**). The detection of the *h-orf* in hermaphrodite gonads, a mitochondrial gene specific to hermaphroditic freshwater mussel species (Breton et al., 2011), supports the hypothesis that this gene could have an essential role in the development of hermaphrodites in freshwater mussels (Breton et al., 2011; Mitchell et al., 2016). Similarly, the detection of genes involved in ubiquitination in hermaphrodite gonads reinforces the hypothesis that this process could be involved in mitochondrial inheritance and sex determination in bivalves (Ghiselli et al., 2012; Capt et al., 2018; Punzi et al., 2018). However, the few annotated genes in **Table 2** (i.e. only 4% of the sex-specific genes were annotated and many of these were uncharacterized proteins) clearly point to the need for further studies of these sex-specific factors to elucidate the molecular mechanisms of sex determination and mitochondrial inheritance in freshwater mussels.

**Table 2:** Sex-specific genes in hermaphrodites, males and females.

Unigene	Annotation (sprot)	e-value	Description	FPKM H (Mean)
c404212_g1_i1	F4ZG36_9BIVA	2,00E-60	H-orf protein	8565.0
c180137_g1_i1	PAR14_HUMAN	2,00E-12	Poly [ADP-ribose] polymerase 14	1295.3
c1018548_g1_i1	DCXR_BOVIN	1,00E-44	L-xylulose reductase	503.3
c168047_g2_i2	PAR14_MOUSE	4,00E-15	Poly [ADP-ribose] polymerase 14	129.0
c213214_g2_i1	POL2_DROME	1,00E-11	Retrovirus-related Pol polyprotein from transposon 297	57.7
c304119_g1_i1	UBIQ_WHEAT	1,00E-08	Ubiquitin	43.7
c703049_g1_i1	BIRC1_HUMAN	9,00E-10	Baculoviral IAP repeat-containing protein 1	39.7
c231908_g1_i1	RN213_MOUSE	4,00E-09	E3 ubiquitin-protein ligase RNF213	25.0
c226075_g1_i1	PIF3_TRYB2	3,00E-07	ATP-dependent DNA helicase PIF3	18.0
c763331_g1_i1	NTF2_ARATH	5,00E-40	Nuclear transport factor 2	16.0
c131247_g1_i1	PLXA3_MOUSE	2,00E-07	Plexin-A3	11.0
Unigene	Annotation (sprot)	e-value	Description	FPKM M (Mean)
c168636_g1_i1	W4Y5B2_STRPU	1,00E-19	Uncharacterized protein	111.8
c230214_g5_i1	G3TY18_LOXAF	1,00E-52	Uncharacterized protein	57.8
c148972_g1_i1	D7GXV5_TRICA	3,00E-06	Putative uncharacterized protein	55.0
c159235_g1_i1	V3ZGW1_LOTGI	4,00E-07	Uncharacterized protein	19.5
c148972_g2_i1	W4Z2T5_STRPU	3,00E-10	Uncharacterized protein	18.3
c150368_g1_i2	K1PNI5_CRAGI	1,00E-06	Putative ankyrin repeat protein L93	24.3
c219930_g7_i1	W4YBE1_STRPU	7,00E-10	Uncharacterized protein	60.5
Unigene	Annotation (sprot)	e-value	Description	FPKM F (Mean)
c209524_g1_i6	ATS12_MOUSE	2,00E-11	A disintegrin and metalloproteinase with thrombospondin motifs 12	120.0
c227946_g1_i1	KY_MOUSE	2,00E-28	Kyphoscoliosis peptidase	92.75
c179701_g1_i2	UPSA_UNIPI	2,00E-73	Upsalin	90.25
c178426_g1_i2	PLC_HALLA	7,00E-12	Perlucin	85.5
c136248_g1_i1	ZNF80_PANTR	2,00E-19	Zinc finger protein 80	72.75
c155883_g1_i1	ISL2_CHICK	3,00E-06	Insulin gene enhancer protein ISL-2	52.75
c137229_g1_i1	A4_RAT	8,00E-10	Amyloid beta A4 protein	40.75
c222051_g4_i1	PCF11_HUMAN	3,00E-17	Pre-mRNA cleavage complex 2 protein Pcf11	30.5
c119723_g1_i2	SNAI1_XENLA	2,00E-25	Protein snail homolog Sna	24.75
c398528_g1_i1	LHX6_MOUSE	2,00E-11	LIM/homeobox protein Lhx6	11.0

As a second step to unravel potential candidate genes involved in sex determination and/or DUI in *Utterbackia* spp, we searched for the 20 most up-regulated differentially expressed genes, based on their log2 Fold-Change (log2FC) values, in each pairwise-comparison; Male–Female (MF), Hermaphrodite–Male (HM) and Hermaphrodite–Female (HF) (**Supplementary Table S1**). For the MF comparison, our results were highly similar to those obtained in our previous study comparing male and female *U. peninsularis* gonadal transcriptomes (Capt et al., 2018), with up-regulated genes in males including factors involved in spermatogenesis, sperm motility and ubiquitination processes, and up-regulated genes in females including C-type lectin domains, which are known to be involved in gamete recognition and polyspermy blocking during fertilization. One particular new female-biased gene found in the present study was *Temptin* (c175440_g1_i1; up-regulated mostly in females, and also to a lesser extent in hermaphrodites compared to males [MF; log2FC = －14.7, HM; log2FC = +11.3, HF; log2FC = －3.4]). Temptin is a water-borne protein pheromone that provokes stimulation of mating behavior in some mollusk species [e.g. the sea slug *Aplysia* sp. (Cummins et al., 2004) and the gastropod *Biomphalaria glabrata* (Pila et al., 2017)]. Because pheromones are thought to play a critical role in triggering spawning in bivalves (e.g. Rice et al., 2002), it is possible that such a pheromone produced by eggs could stimulate male mussels to spawn and/or attract sperm in marsupia gill chambers where eggs are fertilized in freshwater mussels (Haag, 2012).

Several top DEGs identified in the MF comparison were also present in hermaphrodites (i.e. many up-regulated genes in the hermaphrodites compared to females were “male-type” genes such as testis-specific kinases, whereas many up-regulated genes in the hermaphrodites compared to males were “female-type” genes such as C-type lectin domains and *Temptin*). These results suggest similar factors involved in gonadal differentiation between the gonochoric *U. peninsularis* and hermaphrodite *U. imbecillis*.

To decipher potential candidate genes involved in mitochondrial inheritance in *Utterbackia* spp, we clustered all the genes found up-regulated in hermaphrodites compared to males and females grouped together (i.e. SMI vs. DUI). Only annotated genes were kept, and if a similar annotation was also found in male- or female-specific DEGs, it was discarded from the list. This step allowed us to focus on genes with a unique role in hermaphrodites, thus potentially revealing genes specific to SMI of mitochondria (**Supplementary Table S2**). We found 167 genes specifically over-expressed in hermaphrodite gonads. Of these, one unigene was involved in autophagy, seven in nuclease activity and six were involved in polymerase activity, representing three gene ontology categories previously described as being linked to mitochondrial inheritance strategies (Punzi et al., 2018). A similar pipeline was applied for males to find potential candidates directly linked to DUI and retention of sperm mitochondria in male gonads (**Supplementary Table S2**). We found 153 genes specifically over-expressed in males. One of them has a role in methylation events and another in ubiquitination activity. All of these results are discussed in the section “candidate genes involved in mitochondrial inheritance”.

### Gene Ontology (GO) and Enrichment Analyses

**Figure 3:**
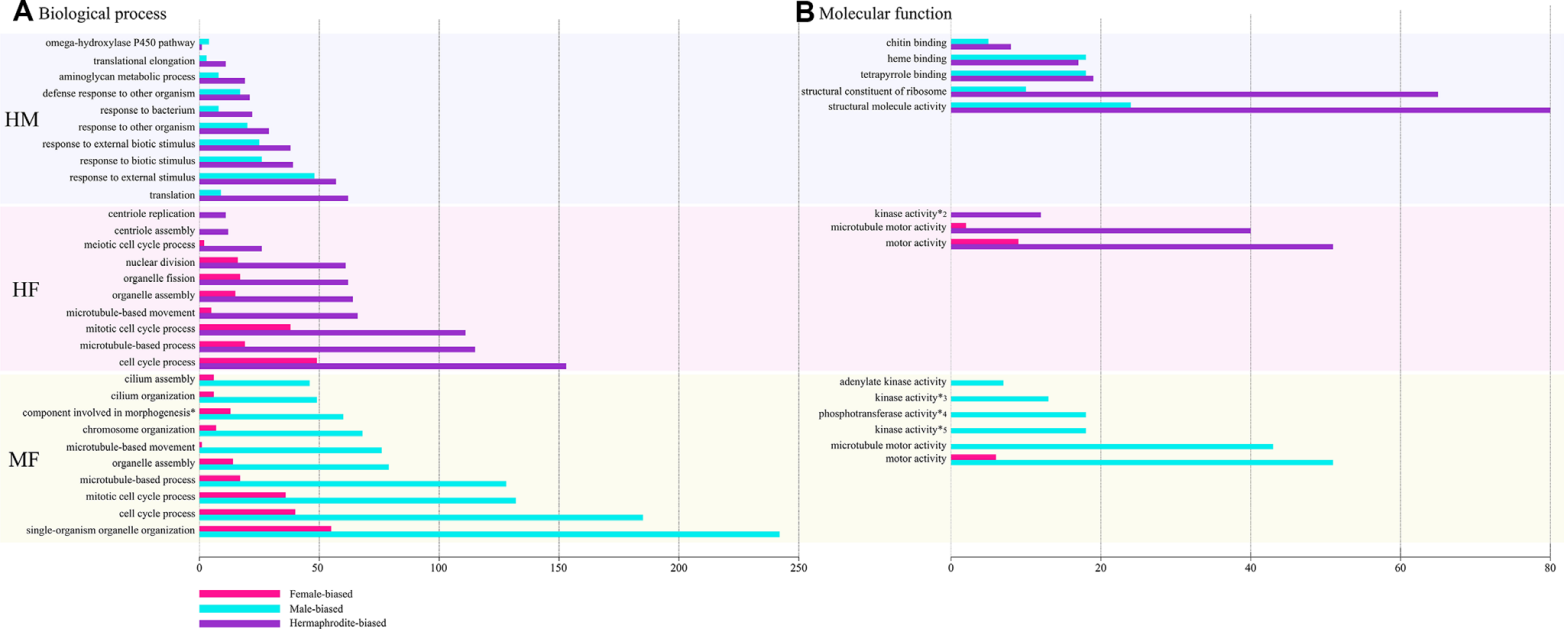
Top ten of most significantly enriched Gene Ontology (GO) categories in each comparison; hermaphrodite–male (HM), hermaphrodite–female (HF), and male–female (MF), involved in both biological processes **(A)** and molecular functions **(B)**. Differentially expressed genes (DEGs) numbers involved in each GO categories are shown by x-axis; female-biased genes are illustrated in pink, male-biased in blue and hermaphrodite-biased in purple. Complete GO terms are written here, *; cellular component assembly involved in morphogenesis, *^2^; nucleobase-containing compound kinase activity, *^3^; nucleoside diphosphate kinase activity, *^4^; phosphotransferase activity, phosphate group as acceptor, *^5^; nucleobase-containing compound kinase activity.

**Figure 3** shows the top ten enriched GO (biological processes and molecular function) categories for each MF, HM, and HF pairwise comparison. For the complete GOseq results, see **Supplementary Table S1**. Overall, GOseq analyses between males and females revealed 110 gene ontology categories significantly enriched out of 621 listed. HF and HM comparisons showed reduced numbers of enriched categories, with 41 (out of 526) and 25 (out of 456), respectively (**Table S1**). The top ten enriched categories were similar for MF and HF comparisons, potentially revealing male gonad specific processes and functions, and included microtubule dynamics (GO:0007017, GO:0007018, GO:0005815, GO:0005874, GO:0044450) and mitotic events (GO:190304, GO:0007067) (**Figure 3**). Microtubule dynamics is important in spermatogenesis and male germ cell development by supervising the division of sperm cells and by modulating sperm shape and motility, leading to correct male fertility (O’Donnell et al., 2012; O’Donnell and O’Bryan, 2014). The up-regulation of microtubule dynamic process in males and hermaphrodites compared to females is thus not surprising. Similar results were also found in previous studies comparing male and female gonad transcriptomes in gonochoric bivalves (Ghiselli et al., 2012; Zhang N et al., 2014; Tong et al., 2015; Shi et al., 2015; Li et al., 2016; Capt et al., 2018). In species with DUI, a role of microtubules in sperm mitochondria aggregation in male gonads has also been suggested (Obata and Komaru, 2005; Ghiselli et al., 2011; Milani et al., 2016), but this still remains to be confirmed. In the HM comparison, over-represented GO categories mostly belonged to response to biotic and external stimuli (GO:0009605, GO:0009607, GO:0043207, GO:0051707, GO:0009617) and ribosome cellular component and activity (GO:0005840, GO:0030529, GO:0044391, GO:0022625, GO: 0015934, GO:0003735). Up-regulation of these GO categories in hermaphrodites could be related to some epigenetic mechanisms with key roles in the differentiation of gametes in hermaphroditic individuals (e.g. Gomot and Griffond, 1993).

### Candidate Genes Involved in Sex Determination

Our approach to identify candidate genes involved in sex determination consisted of searches in the GO categories, terms that contained ‘reproduction’ and ‘sex’. In addition, candidate genes previously identified as sex-determining genes in the literature in bivalves and in model organisms *C. elegans*, *Drosophila melanogaster* and *Mus musculus* were searched in differentially expressed genes (**Table 3**; see also Capt et al., 2018). Of the 26 key sex-determining pathway genes examined, putative homologs were found for 15 genes or gene families in freshwater mussel species (**Table 3**). Based on log2FC values, three main factors were up-regulated in males and hermaphrodites compared to females, i.e. *Dsx/Dmrt1/mab3, Sry* and *Fem1c*, all of which are all known to play an important role in male sex determination (Wallis et al., 2008; Kopp, 2012). In contrast, *Foxl2*, which is a well-known gene implied in oogenesis differentiation in a large variety of species (Uhlenhaut and Treier, 2006), was up-regulated in both females and hermaphrodites when compared to males. Overall, our results are quite similar to what has been observed in previous transcriptomic studies of freshwater mussels or other bivalve species (Capt et al., 2018). One novelty in the present study is the discovery of a highly up-regulated gene in females and hermaphrodites compared to males; *Zglp1*, which codes for a GATA-4 protein and has a role in early-gonadal differentiation in mammals (Viger et al., 1998).

**Table 3:** Candidate genes involved in sex determination pathway in freshwater mussels and other bivalves.

Gene symbol	Name	Gonochoric FWM species	Hermaphroditic FWM species	Other gonochoric bivalves species
*Amh, Amrh2 (Mmu)*	Anti-Mullerian hormone and receptor type 2			
*Bar-1 (Cel), Arm (Dme), Ctnnb1 (Mmu)*	Beta catenin			
*Cbx2 (Mmu)*	Chromobox 2			
*Doa (Dme)*	Darkener of apricot			
*Dsx (Dme), Dmrt1 (Mmu), Mab-3 (Cel)*	Doublesex- and mab-3-related transcription factor 1			
*Fog-1,-2,-3 (Cel, Dme, Mmu)*	Feminization of germline			
*Fem-1 (Cel, Dme), Fem1b (Mmu)*	Feminization of XX and XO animal			
*FoxL2 (Mmu)*	Forkhead box L2			
*Fru (Dme)*	Fruitless			
*Zglp1 (Mmu)*	GATA-type zinc finger protein 1			
*Her-1 (Cel), Her (Dme)*	Hermaphrodization of XO animals			
*Nr0b1 (Mmu)*	Nuclear receptor subfamily 0, group B, member 1			
*Rspo1 (Mmu)*	R-spondin 1			
*Rnt-1 (Cel), Run (Dme)*	Segmentation protein Runt			
*Sdc (Cel, Dme, Mmu)*	Sex determination and Dosage Compensation defect			
*Sxl, fl(2)d (Dme)*	Sex lethal, female-lethal(2)d			
*Sry/Sox30/SoxH (Mmu)*	Sex-determining region of chromosome Y, SRY (sex determining region Y)-box 30			
*Sis-a (Dme)*	Sisterless			
*Sf1 (Mmu)*	Splicing factor 1			
*Sox9 (Mmu), Sox100B (Dme)*	SRY (sex determining region Y)-box 9, SRY-box 100B			
*Gata4 (Mmu)*	Transcription factor GATA-4			
*Tra-1 -2 -3 (Cel), Tra2 (Dme)*	Transformer			
*Wt1 (Mmu)*	Wilms tumor 1 homolog			
*Wnt4 (Dme, Mmu)*	Wingless-type MMTV integration site family, member 4			
*Xol-1 (Cel)*	Xol-1 XO Lethal			

Names in parentheses indicate the species in which each gene has been identified (Cel, C. elegans; Dme, Drosophila melanogaster; Mmu, Mus musculus) (the list was first provided by Zhang N et al., 2014); Gonochoric FWM (freshwater mussel) species include Venustaconcha ellipsiformis and Utterbackia peninsularis (Capt et al., 2018; present study) and Hyriopsis schlegelii (Shi et al., 2015); Hermaphroditic species include Utterbackia peninsularis (present study); Other gonochoric bivalve species include the oysters Crassostrea gigas and C. honkongensis, the scallop Patinopecten yessoensis and the marine clams Ruditapes philippinarum and R. decussatus (see Capt et al., 2018); pink box indicates female-specific or female-biased expression; pale pink box indicates hermaphrodite-biased expression compare to males; blue box indicates male-specific or male-biased expression; pale blue box indicates hermaphrodite-biased expression compare to female; grey box indicates presence in the transcriptome but not differentially expressed.

### Candidate Genes Involved in Mitochondrial Inheritance

To identify candidate genes involved in mitochondrial inheritance, we used the approach proposed by Punzi et al. (2018), i.e. to compare our SMI (*U. imbecillis*) vs. our DUI (*U. peninsularis*) species, while considering presence and transcription patterns of proteins belonging to pathways known to be involved in mitochondrial inheritance in animal species. We thus analyzed transcripts belonging to the GO categories “nucleases/polymerases,” “autophagy and mitophagy,” and “ubiquitination pathway” and compared our results with those obtained for the marine clam SMI species *Ruditapes decussatus* and the DUI species *R. philippinarum* (Ghiselli et al., 2012; Milani et al., 2013b; Punzi et al., 2018). As mentioned above, we also searched the hermaphrodite-specific and male-specific DEGs for genes related to these categories. Furthermore, because autophagy and mitophagy pathways rely on evolutionarily conserved core components, we used the compiled lists of orthologs related to these processes generated previously (Lee and Han, 2017; Punzi et al., 2018) and used successfully by Punzi et al. (2018) for the bivalve species *Ruditapes philippinarum* and *R. decussatus*, to search for their presence in *Utterbackia* spp based on our annotations. Finally, we discuss transcripts belonging to “methylation GO categories” because our previous study identified this process as potentially important in influencing mtDNA inheritance and/or regulating sex determination and differentiation in bivalves (Capt et al., 2018).

### Ubiquitination-Related Factors

The proposed mode of action for ubiquitination in SMI is to tag paternal mitochondria for degradation during spermatogenesis and/or after fertilization (Sato and Sato, 2017; Punzi et al., 2018), and a modification of this mechanism has been proposed to be responsible for DUI of mitochondria in bivalves (Ghiselli et al., 2012; Milani et al., 2013b). For example, three main male-biased ubiquitination-related factors [*birc —* baculoviral IAP repeat-containing 4 (inhibitor of apoptosis), *psa* — proteasome subunit alpha 6, and *anubl* — AN1 zinc finger ubiquitin-like domain] have previously been hypothesized to be responsible for the maintenance/degradation of spermatozoon mitochondria during embryo development of the DUI species *R. philippinarum* (Ghiselli et al., 2012; Milani et al., 2013b). These three factors were also reported as male-biased in the freshwater mussel *Utterbackia peninsularis* (Capt et al., 2018). In the present study, a transcriptional bias (up-regulation) for ubiquitination-related genes is thus expected in males and hermaphrodites compared to females. Our GOseq results revealed seven ubiquitination-related categories enriched (but not significantly) in DUI males (**Table S3**). The only DEG corresponding to our expectation was annotated as protein MAD2A and is involved in mitotic events (**Tables S2** and **S3**). In our complete list of DEGs, several baculoviral IAP repeat-containing protein-coding genes were found, including male-biases *birc* genes such as *birc-5*, which was up-regulated in males but also in hermaphrodites when compared to females (**Table 4**). *Anubl1* was also found up-regulated in males and hermaphrodites compared to females (**Table 4**). According to previous studies (Milani et al., 2013b; Capt et al., 2018), *birc* could be an additional mitochondrial tag in DUI species that would protect the M midpiece mitochondria from degradation and *anubl1* could be a factor tagging sperm mitochondria that differentiate them from egg mitochondria in DUI embryos. Our results indeed suggest that these genes are mainly male-related but their link to paternal mitochondrial inheritance remains to be clearly demonstrated since they were also found in hermaphrodite and female individuals, both of which lack the M mtDNA. One other ubiquitination-related factor up-regulated in males and hermaphrodites compared to females was the F-box only protein 39 (**Table 4**). F-box proteins are important components of the protein degradation pathway involving ubiquitination (Ho et al., 2006). Hermaphrodite-specific ubiquitination-related genes were also found (**Table S2**), reinforcing the hypothesis that this process might be involved in mitochondrial inheritance in freshwater mussels. Finally, nine unigenes were annotated with *psa*, but only one of them was overexpressed in males and hermaphrodites compared to females (**Table 4**), supporting the hypothesis that subunits alpha of the proteasome may have a role in mitochondrial inheritance and/or male sex determination or differentiation in bivalve species (Milani et al., 2013b).

**Table 4:** Putative candidate genes involved in mitochondrial inheritance in freshwater mussels.

Contig ID	Gene name	HM	HF	MF	GO category
c211580_g1_i1	*Birc5*	–	(+1.7)	(+2.0)	Ubiquitination
c223534_g1_i1	*Anubl1*	–	(+5.0)	(+6.2)	Ubiquitination
c211818_g1_i1	*FBX039*	–	(+10.2)	(+11.0)	Ubiquitination
c224112_g6_i2	*Psa*	–	(+1.5)	(+2.0)	Ubiquitination
c154451_g1	Nuclease EXOG, mitochondrial	(+5.8)	(+7.6)	–	Nuclease, polymerase
c226166_g1_i2	TANK-binding kinase 1	(+1.8)	(+1.4)	–	Autophagy, mitophagy
c235196_g1_i1	Ras-like GTP-binding protein YPT1	(+7.6)	(+4.4)	–	Autophagy, mitophagy
c231455_g1_i6	DNMT1	–	(+2.9)	(+3.8)	Methylation

The annotation format was provided by the Uniprot database HM, HF and MF respectively indicate hermaphrodites vs. males, hermaphrodites vs. females and males vs. females comparisons, log2 fold change values (obtained from DESEq2 software) are in parentheses, a positive value means that the expression is biased towards the first sex in the comparison, a negative value indicates that the expression is biased towards the second sex in the comparison.

### Nucleases and Polymerases

The proposed mode of action of nucleases and polymerases is to degrade mtDNA during spermatogenesis to prevent transmission of paternal mtDNA (Sato and Sato, 2017; Punzi et al., 2018). For example, endonuclease G (*endoG* gene) has been shown to eliminate paternal mitochondrial nucleoids during spermatozoid production in *D. melanogaster*, thus resulting in SMI of mitochondria (Chan and Schon, 2012;DeLuca and O’Farrell, 2012). *Tamas*, a gene that codes for one subunit of the mtDNA polymerase (Pol γ-α), has also been identified as a key factor in this process (Yu et al., 2017). Here, a transcriptional bias for such genes (an up-regulation) is expected in SMI hermaphrodites compared to DUI males and females. Our GOseq results and hermaphrodite-specific DEGs revealed 13 genes that corresponded to this situation (**Supplementary Table S3**), including ten genes coding for Gag or Pol proteins. These genes represent signatures of retrovirus and transposon activity, suggesting a key role for these proteins in hermaphrodite gonads. One promising gene overexpressed in hermaphrodites compared to DUI males and females was *exoG* (**Table 4**), which is a paralog of *endoG* described in higher eukaryotes (Cymerman et al., 2008). It must be noted that *endoG* was also found but was up-regulated in males and females compared to hermaphrodites (data not shown). We propose that *exoG* could represent a potential candidate involved in SMI of mitochondria in hermaphroditic freshwater mussels. Like in *Drosophila*, paternal mtDNA could be mostly degraded by a nuclease-mediated mechanism during the process of spermatogenesis, and paternal mitochondrial structure could be degraded by ubiquitination-related mechanisms after fertilization (Sato and Sato, 2017).

### Autophagy and Mitophagy

The proposed mode of action of autophagy and mitophagy is to degrade mitochondria during spermatogenesis and/or after fertilization to prevent transmission of paternal mtDNA (Sato and Sato, 2017; Punzi et al., 2018). If these processes are involved in mitochondria degradation during spermatogenesis to maintain SMI, it is expected that they will be up-regulated in SMI hermaphrodites compared to DUI males and females. In addition with GO terms searches, and because autophagy and mitophagy are well-conserved processes, specific autophagy-orthologs listed in Lee and Han (2017) and Punzi et al. (2018) were also searched in our DEGs. Only two genes involved in mitophagy/autophagy processes were indeed up-regulated in the SMI species (**Table 4** and **Supplementary Table S3**): (1) TANK-binding kinase 1, a protein involved in mitophagy regulation in the PINK1-Parkin pathway (Heo et al., 2015), and (2) Ras-like GTP-binding protein YPT1, which is linked to organelle inheritance in yeast (Lewandowska et al., 2013). Combined with *exoG*, these proteins could belong to the pre-fertilization mechanisms involved in the strict maternal inheritance of mitochondria in hermaphroditic freshwater mussels.

On the other hand, if autophagy and mitophagy are involved in sperm mitochondria degradation after fertilization, it is expected that they will be over-expressed in DUI females and SMI hermaphrodites compared to DUI males. None of our data fulfilled these criteria, a result similar to what was found in *Ruditapes* spp. (Punzi et al., 2018).

### Methylation

As previously suggested, methylation could play an important role in mitochondrial inheritance and/or sex determination in animal species, including freshwater mussels (Liu et al., 2015; Capt et al., 2018). In *Chlamydomonas reinhardtii*, there are two mating types (mt+ and mt－), and uniparental inheritance of chloroplast DNA (cpDNA) is achieved by active elimination of the mt-cpDNA, with a possible role of DNA methylation in guaranteeing strict uniparental inheritance by enhancing the replication of the mt+ cpDNA (while unmethylated cpDNAs is being degraded) (Nishimura, 2010). If methylation is involved in DUI of mitochondria in bivalve species, with methylated paternal mtDNA being protected from degradation, we could expect an up-regulation of genes related to this process in DUI males compared to SMI hermaphrodites and DUI females. Our GOseq results and male-specific DEGs revealed two annotated genes fulfilling these criteria, i.e. two methyltransferases, *protein arginine N methyltransferase 1/6* that methylates proteins and *putative methyltransferase C9orf114 homolog isoform X1* that methylates RNA (**Supplementary Table S3**). Interestingly, the methyltransferase DNMT1, for which a mitochondrial isoform has been described (Maresca et al., 2015), was found to be overexpressed in males and hermaphrodites compared to females. If DNMT1 is differentially expressed in males and hermaphrodites’ male-parts gonads, it is not related with the assumption that paternal methylated mtDNA would be protected from nucleases, except if another mechanism in SMI hermaphrodites can overcome this event, for example through the endonuclease activity of *exoG*. Further studies will be necessary to unravel this possibility.

## Conclusion

In this study, we reported the transcriptome of male, female, and hermaphrodite gonads of two freshwater mussel species, i.e. the gonochoric, DUI species *Utterbackia peninsularis* and the hermaphrodite, SMI species *U. imbecillis*. The results were used to generate a “hybrid transcriptome,” which allowed analyzing differential gene expression among the three sexes. Our results were also compared to published transcriptomes of bivalve species showing different mitochondrial transmission modes (DUI and SMI). This approach permitted us to highlight sex-specific and sex-biased expression patterns, as well as expression patterns associated to SMI or DUI within bivalves having alternative reproductive and mitochondrial transmission modes. Our results revealed similar combinations of genes with conserved sex-linked roles in gonochoric and simultaneous hermaphrodite freshwater mussel species and suggested that steps of the sex-determination pathway are conserved in distantly-related bivalve species. Our results also revealed new candidate genes showing potential roles for SMI or DUI of mitochondria in bivalves, including nucleases and factors involved in autophagy/mitophagy and methylation. Further investigation of these genes will help to unravel the mechanisms underlying mitochondrial inheritance and sex determination in bivalves.

## Data Availability

The datasets generated for this study can be found in National Centre for Biotechnology Information (NCBI) Sequence Read Archive (SRA) database, SRR8782945-7.

## Author Contributions

CC, SR, and SB designed experiments. NJ conducted sampling. CC performed laboratory experiments. CC and SR analyzed data. CC, SR, NJ, DS and SB wrote the manuscript. SB conceived and managed the project. All authors read and approved the final version of the manuscript.

## Funding

Financial support for this study has been provided by Natural Sciences and Engineering Research Council Discovery Grant awarded to S Breton [grant number RGPIN/435656-2013]. Any use of trade, firm, or product names is for descriptive purposes only and does not imply endorsement by the U.S.

## Conflict of Interest Statement

The authors declare that the research was conducted in the absence of any commercial or financial relationships that could be construed as a potential conflict of interest.
