# Supplementary figures and images for: Putative Mitochondrial Sex Determination in the Bivalvia: Insights From a Hybrid Transcriptome Assembly in Freshwater Mussels
Source: Front Genet. 2019 Sep 13;10:840. doi: 10.3389/fgene.2019.00840 (PMC6754070; doi:10.3389/fgene.2019.00840)

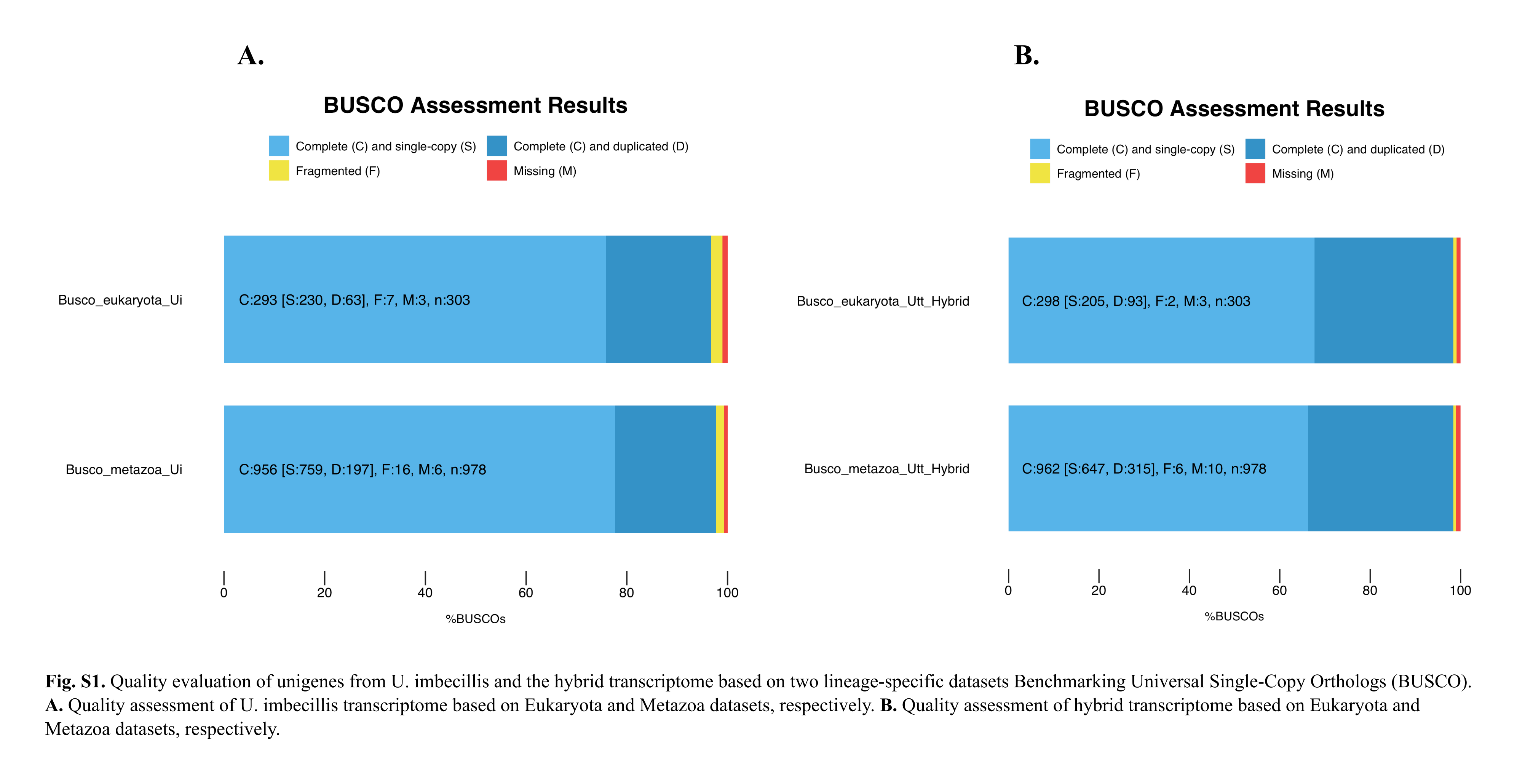

Supplement: Supplementary file 4 [file Image_1.jpeg]
